# Idiopathic scoliosis pathogenesis

**DOI:** 10.1186/1748-7161-7-S1-P8

**Published:** 2012-01-27

**Authors:** M Dudin, D Pinchuk

**Affiliations:** 1 Center of Rehabilitation for Children’s Orthopedic Diseases, St.Petersburg, Russia

## 

Based on long-term investigations we have concluded that IS is a compensatory response of the vertebral complex and has several strictly sequential stages:

The first stage – pre-clinical. The usage of physiological kyphosis reserve (kyphosis is being transformed into lordosis).

The second stage – sub-clinical. Elimination of the “excess” length of osseous vertebra column owing to its torsion around relatively short medulla spinalis: frontal axis of shoulder (thoracic) girdle loses its parallelism relatively to the same axis of pelvis. “Operate” muscles-rotators of caudal zone.

The third stage – clinical. Generated at the second stage stable shoulder (thoracic) girdle torsion is eliminated thanks to contra-lateral muscles-rotators in cranial zone and at the level where the process begins is left the most rotated vertebra that comes to be the top of the functional scoliosis. From that standpoint can be explained the well known fact of increased electro-activity of paravertebral muscles on the convex side of the scoliotic curve.

The fourth stage – vicious circle. Generated at the third stage functional frontal curve disturbs vertebra column biomechanics and creates all conditions for realization of Huter-Volkman’s law. The order of the following actions is evident: asymmetric pressure brings to development of vertebrae bodies wedge-shape, which sequentially, increases the frontal curve, etc. During examination of such patients the physician becomes the witness of typical IS development – tragedy, where the stage is vertebral complex.

This opens up new horizons in treatment of scoliosis.

